# Synchronous development of intrahepatic cholangiocarcinoma and hepatocellular carcinoma in different sites of the liver with chronic B-viral hepatitis: two case reports

**DOI:** 10.1186/1756-0500-6-520

**Published:** 2013-12-07

**Authors:** Kyu Sik Jung, Kyeong Hyeon Chun, Gi Hong Choi, Hyae Min Jeon, Hye Sun Shin, Young Nyun Park, Jun Yong Park

**Affiliations:** 1 Department of Internal Medicine, Yonsei University College of Medicine, 50 Yonsei-ro, Seodaemun–gu, Seoul 120-752, Korea; 2 Department of Surgery, Yonsei University College of Medicine, 50 Yonsei-ro, Seodaemun–gu, Seoul 120-752, Korea; 3 Department of Pathology, Yonsei University College of Medicine, 50 Yonsei-ro, Seodaemun–gu, Seoul 120-752, Korea; 4 Institute of Gastroenterology, Yonsei University College of Medicine, 50 Yonsei-ro, Seodaemun–gu, Seoul 120-752, Korea; 5 Liver Cancer Special Clinic, Severance Hospital, 50 Yonsei-ro, Seodaemun–gu, Seoul 120-752, Korea

**Keywords:** Hepatocellular carcinoma, Intrahepatic cholangiocarcinoma, Double primary liver cancer, Hepatitis B virus, Chronic liver disease

## Abstract

**Background:**

Synchronous development of primary hepatocellular carcinoma and intrahepatic cholangiocarcinoma has been reported rarely. In literature review, there have been only 35 reported cases of synchronous hepatocellular carcinoma and intrahepatic cholangiocarcinoma, and most of these tumors developed in patients with hepatitis C-related liver cirrhosis. Here, we present synchronous development of hepatocellular carcinoma and intrahepatic cholangiocarcinoma in two patients with chronic B-viral hepatitis.

**Case presentation:**

Two patients with chronic hepatitis B were referred to our hospital due to a hepatic mass. Patient 1 had a 6.4 cm multinodular hepatic mass in the left lobe and a small nodule in the right lobe. Patient 2 had a 4.3 cm hypervascular mass in the right lobe and a 1.1 cm nodule in the left lobe. The pre-operative diagnosis of both cases was hepatocellular carcinoma with metastatic nodule, however, surgical resection pathology revealed that hepatocellular carcinoma and intrahepatic cholangiocarcinoma existed independently in the other side of the liver in both cases. Additionally, the background liver histology of both cases was hepatitis B-related chronic hepatitis without cirrhotic change.

**Conclusion:**

Our cases suggest that hepatitis B virus infection can also predispose to development of double liver cancers.

## Background

Although hepatocellular carcinoma (HCC) and intrahepatic cholangiocarcinoma (ICC) are the two major forms of primary liver cancer, coincidence of both in a single patient is very rare [1,2]. Moreover, synchronous development of double cancer is exceedingly rare, reported in only 35 cases worldwide [3-6]. Even though the clinicopathological features of the phenomenon have not been clearly investigated, previous studies suggested a relationship between hepatitis C virus (HCV) and the double primary liver cancer [3,4]. These results could be explained by the fact that many of these cases were reported in Japan, where HCV infection is endemic, and HCV-related cirrhosis is associated with carcinogenesis, giving rise to both HCC and ICC [7,8]. In contrast of HCV-infections, the relationship between hepatitis B virus (HBV) infection and double primary tumor has not been clarified yet, although it is known that HBV is an important risk factor for development of HCC and ICC [9,10]. Here, we present two chronic hepatitis B cases that resulted in synchronous development of HCC and ICC in separate hepatic sites.

## Case presentation

### Case 1

A 66-year-old woman with chronic hepatitis B was referred to our clinic due to a hepatic mass. The patient had no other apparent conditions. Laboratory tests, including platelet counts and liver function, were normal. Hepatitis B surface antigen (HBsAg) was positive and hepatitis B e antigen (HBeAg) was negative. The serum hepatitis B virus deoxyribonucleic acid (HBV-DNA) level was 2,490 IU/ml. Anti-HCV antibody was negative. Of the tumor markers tested, alpha-fetoprotein (AFP) was 3.54 IU/ml, protein induced vitamin K antagonist (PIVKA-II) was 22 mAU/ml, carcinoembryonic antigen (CEA) was 4.28 ng/ml, and carbohydrate antigen 19–9 (CA19-9) was 0.1 U/ml. She was a social-drinker, and had no metabolic impairment.

**Figure 1:**
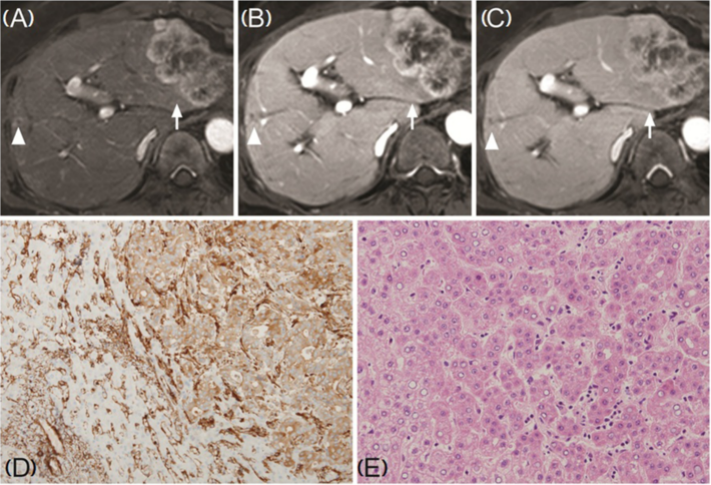
**Dynamic magnetic resonance imaging (MRI) of the liver (A-C, 3D T1-weighted gradient echo image) and histology of surgical specimens (D, E).** A 6.4 cm-sized main mass (*arrow*) showed heterogeneous rim-enhancement throughout arterial **(A)**, portal **(B)**, and delayed phase **(C)**, and small nodule (*arrowhead*) in the right lobe also showed rim enhancement. **D**: A moderately differentiated cholangiocarcinoma (immunohistochemical staining of Filamin-A). **E**: An Edmondson-Steiner grade I, trabecular type hepatocellular carcinoma. (H&E, D: ×200, E: ×100).

Computed tomography (CT) and dynamic magnetic resonance imaging (MRI) revealed a 6.4 cm multinodular hepatic mass with heterogeneous arterial rim enhancement in the left lobe (S2 and 3), and a small nodule in the right lobe (S7) (Figure 1A-C). The larger mass was suspected to be an HCC and the smaller mass was suspected to be a metastatic nodule, but due to the atypical radiological findings of the main tumor and the normal tumor marker levels, we were unable to confirm the preoperative diagnosis. The tumors were resected via left lateral sectionectomy (S2 and S3) and wedge resection of S7.

Unexpectedly, the pathological examination revealed that the main mass in the left lobe was moderately differentiated ICC (Figure 1D, positive immunohistochemical staining of Filamin-A) and the small nodule in the right lobe was an HCC of Edmondson-Steiner grade I (Figure 1E). According to histopathological classification proposed by Komuta *et al*., the tumor in the left lobe was pure mucin-producing adenocarcinoma [11]. Additionally, it was determined that the ICC had metastasized to two lymph nodes. Histology of the hepatic parenchyma revealed chronic hepatitis caused by hepatitis B virus with minimal lobular activity and Metavir grade 1 fibrosis.

For one month following surgical resection, the patient was treated with adjuvant chemotherapy using gemcitabine hydrochloride, after which she underwent six cycles of chemotherapy. At seven months post resection, there was no evidence of recurrence.

### Case 2

**Figure 2:**
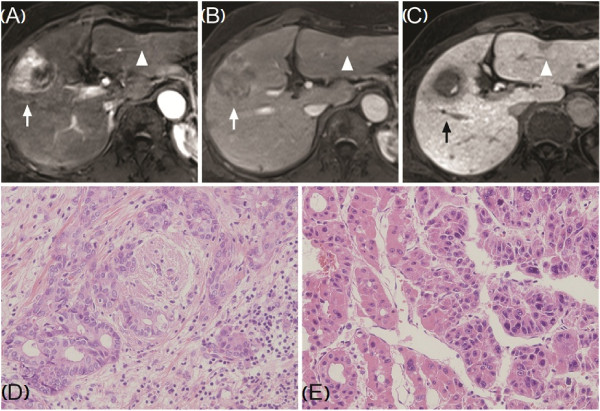
**Dynamic magnetic resonance imaging (MRI) of the liver (A-C, T1-weighted spoiled gradient echo image), and histology of surgical specimens (D-E).** The right lobe mass (*arrow*) appears as a high-intensity lesion during the arterial phase **(A)** with delayed wash-out (**B**: portal phase, **C**: delayed phase). The satellite lesion (*arrowhead*) in the left lobe has peripheral signal enhancement and slightly higher signal in the central area during the arterial and portal phases **(A, B)**, and low-intensity signal during delayed phase **(C)**. **D**: Frozen section pathology, a well-differentiated cholangiocarcinoma. **E**: Edmondson-Steiner grade II/III, trabecular- and pseudoglandular-type hepatocellular carcinoma. (H&E, D, E: ×200).

A 68-year-old woman with chronic B viral hepatitis was referred to our hospital due to a hepatic mass. She was asymptomatic and her laboratory tests were normal. HBsAg was positive and HBeAg was negative. The serum HBV-DNA level was 217,000 IU/ml. Anti-HCV antibody was negative. Of the tumor markers tested, AFP was 46.54 IU/ml and PIVKA-II was more than 2,000 mAU/ml. The CEA and CA19-9 level was 1.03 ng/ml and 12.8 U/ml, respectively. She was a social-drinker, and had no metabolic impairment. CT and MRI revealed a 4.3 cm hypervascular mass with internal hemorrhage in segment 5 (S5), and the radiological appearance of the tumor suggested an HCC. Additionally, a 1.1-cm solid lesion with capsular retraction was identified in the left lobe (S3) and suspected as a small HCC or a bile duct adenoma (Figure 2A-C). The patient underwent anterior sectionectomy of the right lobe and an S3 segmentectomy. The pathological examination confirmed that the right lobe tumor was an HCC of Edmondson-Steiner grade II/III (Figure 2E). The left lobe nodule was confirmed as a well-differentiated ICC (Figure 2D). ICC in the left lobe was classified into pure mucin-producing adenocarcinoma by histopathological classification. Histology of the hepatic parenchyma revealed chronic hepatitis caused by hepatitis B virus with mild lobular activity and Metavir grade 2 fibrosis. There was no evidence of recurrence at two years following resection.

## Conclusion

The present report describes two cases of synchronous development of HCC and ICC in different liver lobes with hepatitis B virus infection. Although it is extremely rare for the liver to be affected by two types of primary tumors simultaneously, new insights of stem cell-derived carcinogenesis might be able to explain development mechanism of these tumors as they suggested that hepatic progenitor cell (HPC) can differentiate into either hepatocytes or cholangiocytes [11,12]. According this theory, HPC-related tumors can display a whole spectrum of phenotypes with varying hepatocellular and choloangiocellular differentiation characteristics [11]. For instance, when Komuta *et al.* compared gene expression of cholangiolocellular carcinoma, a subtype of ICC, with those of HCC with HPC characteristics, a high homology was demonstrated between two groups and these results suggested that ICC and HCC may share common carcinogenesis [13].

Along with advanced comprehension of carcinogenesis, there have been additional efforts to find clinicopathological factors related with these tumors [2,3]. Previous reports noted a relationship between chronic liver inflammation and multiple primary liver neoplasms, as most cases involved chronic hepatitis or cirrhosis [3,4]. Moreover, these relationships were theoretically supported by recent studies which indicated that chronic liver inflammation played a key role at the molecular level in primary liver cancers, including HCC and ICC [14-16]. Thus, any etiology causing chronic liver inflammation could be a potential risk factor for coincidental liver tumors. Of these, HCV infection is considered to have one of the closest associations with development of double primary tumor of liver [9]. Indeed, chronic hepatitis C infection is a dominant risk factor for ICC as well as HCC, and to-date, more than 70% of cases of multiple liver tumors with HCC and ICC have been detected in livers infected with HCV [7,8].

In contrast to HCV infection, HBV infection has not been associated with the development of synchronous liver tumors, despite being the most common cause of chronic liver disease [17]. Of 35 reported worldwide cases of multiple primary liver tumors, only three patients had HBV infection (8.6%) [3-5]. Especially, in Korea, where the prevalence of HBV infections is considerably high [18], only one case of HBV infection has been reported [5]. However, like HCV infection, HBV infection is a well-known risk factor of HCC development [9,19], and recent studies including one meta-analysis strongly suggest that HBV infection also increases the risk of ICC significantly [10,20-22]. Moreover, recent studies indicated that hepatitis B virus-associated ICC and HCC shared common disease process for carcinogenesis in the cross-sectional design [12]. These results suggest that not only does HBV cause synchronous development of HCC and ICC, but also that the low prevalence of these tumors in HBV infection may be due to underdiagnosis in clinical practice.

Indeed, Inaba *et al*. stated that most cases of multiple liver tumors were diagnosed initially as HCCs, with only about 20% being accurately diagnosed as separate primary liver tumors prior to treatment [3]. There are several possible explanations for this. Firstly, recent improvements in radiologic imaging allow the clinician to diagnose many HCCs without liver biopsy [23]. However, in some cases, ICC can be confused radiologically with HCC, and small tumors or malignant nodules may not resemble a typical HCC or ICC. In case 2 of this report, the main mass had characteristics typical of HCC with increased AFP and PIVKA-II, and the 1.1 cm left lobe mass did not have the typical features of an ICC. Therefore we did not suspect the possibility of synchronous primary tumors. Secondly, paradoxically, it is possible that the clinician’s knowledge of a patient’s HBV or HCV infection could affect the approach to cancer diagnosis, because the clinician may have a preconception of a specific tumor. In case 1 of this report, although the main tumor had ambiguous radiologic morphology and the tumor markers AFP and PIVKA were within normal ranges, our preoperative diagnosis was an HCC. On review of the CT and MRI scans after surgical resection, we admitted that the main tumor had several features of an ICC, but it was still difficult to confirm the diagnosis. In this case, the diagnosis might have been further confused by the knowledge of a higher prevalence of HCC than ICC in HBV patients. Such difficult preoperative diagnosis suggests that there may have been many unproven cases of multiple primary cancers in past cases. Although the prevalence of synchronous primary hepatic tumors is very low, these cases emphasize the importance of a delicate approach to diagnosis of liver cancer. If radiological examination or tumor markers are atypical of a specific cancer, pathological confirmation by liver biopsy should be considered especially in chronic hepatitis patients, even if a certain type of tumor is strongly suspected.

Interestingly, in our cases, the double primary cancer developed from non-cirrhotic liver with HBV-related chronic hepatitis, whereas most of these tumors did from HCV-related cirrhotic livers [3,4]. These results are consistent with the fact that HBV-related HCC could have developed from non-cirrhotic livers and that ICC and HCC share similar mechanism of tumorigenesis in the setting of chronic inflammation [24]. However, the mechanism for developing double liver cancer from liver with HBV infection needs to be further investigated.

As the incidence of synchronous primary liver tumors is extremely low, treatment of these patients is challenging. Although several other methods have been tried, surgical resection is regarded as the treatment of choice because it can provide an accurate diagnosis as well as a chance of cure. But there is no definite guideline after surgical resection, and the postoperative plan should be individualized.

We are aware of the limitations of our study. Most of all, several immunohistochemical markers, which represent human hepatic stem cell or mature cholangiocytes such as epithelial cell adhesion molecule (EpCAM) or secretin receptor, were not available in our cases. Although the novel classification of cholangiocarcinoma proposed by Komuta *et al.*, which is based on histopathological features, might help to identify characteristics of the tumors, these limitations would not allow us to understand carcinogenesis of our cases thoroughly [11,25]. Therefore, application of novel classification of cholangiocarcinoma and appropriate immunohistochemical stain of tumor tissue should be considered in cases with double liver cancer.

In summary, we report here rare cases of synchronous development of HCC and ICC in patients infected B-viral chronic hepatitis. Accurate diagnosis and individualized treatment is important. Further research is needed because knowledge of the biology of these tumors might shed light on the behavior of HCC and ICC and elucidate a more appropriate treatment plan.

## Consent

Written informed consent was obtained from both patients for publication of this case report and accompanying images. A copy of the written consents are available for review by the Editor-in-Chief of this journal.

## Abbreviations

HCC: Hepatocellular carcinoma; ICC: Intrahepatic cholangiocarcinoma; HCV: Hepatitis C virus; HBV: Hepatitis B virus; HBsAg: Hepatitis B surface antigen; HBeAg: Hepatitis B e antigen; HBV-DNA: Hepatitis B virus deoxyribonucleic acid; AFP: Alpha-fetoprotein; PIVKA-II: Protein induced vitamin K antagonist-II; CEA: Carcinoembryonic antigen; CA19-9: Carbohydrate antigen 19–9; CT: Computed tomography; MRI: Magnetic resonance imaging; HPC: Hepatic progenitor cell; EpCAM: Epithelial cell adhesion molecule.

## Competing interests

The authors declare that they have no competing interests.

## Authors’ contributions

KSJ, KHC and HSS collected the clinical data of patients and wrote the manuscript. GHC contributed to the editing and design the article. HMJ and YNP contributed to the analysis and interpretation of pathological data. JYP planned to report the cases and supervised the whole work. All authors gave final approval for publication.
